# What do healthcare workers in elderly care know about occupational health and safety? An explorative survey

**DOI:** 10.1186/s12995-015-0079-0

**Published:** 2015-09-26

**Authors:** Stefanie Schönrock, Anja Schablon, Albert Nienhaus, Claudia Peters

**Affiliations:** University Medical Center Hamburg-Eppendorf (UKE), Institute for Health Services Research in Dermatology and Nursing (CVcare), Martinistrasse 52, 20246 Hamburg, Germany; Department of Occupational Health Research, Institute for Statutory Accident Insurance and Prevention in the Health and Welfare Services (BGW), Hamburg, Germany

**Keywords:** Healthcare workers, Infection prevention, Elderly care, Occupational health

## Abstract

**Background:**

Demographic changes will lead to a growing demand for healthy, motivated healthcare workers (HCW) in the years ahead. Along with well-targeted prevention, knowledge of occupational health and safety and infection precaution is essential for a healthy working life. In this context back-friendly working methods and protection from infectious diseases are necessary in elderly care.

**Methods:**

In 2012, a survey was conducted in nine residential and two semi-residential nursing homes, as well as in one home care service in the Schwerin area of northeast Germany. Four hundred and seventy three HCWs were asked to fill in a questionnaire on what they knew about aspects of occupational health and safety such as vaccinations and preventative measures administered by occupational physicians, hygiene, back-friendly working methods and infection prevention. The statistical evaluation was descriptive, with a comparison between job title. Differences were examined with chi square or Fisher's exact test.

**Results:**

The response rate was 28 % (*n* = 132). The largest group of respondents (36 %) were qualified geriatric HCWs. More than 74 % of employees felt well informed about opportunities for precautionary checks and vaccination by occupational physician, and 93 % utilized these opportunities. When it came to assigning modes of transmission to specific infectious diseases, only 23 % of participants were well informed, and one in three (31 %) care assistants was inadequately informed. Fewer than half of participants could correctly name the indications for hand disinfection. Only 66 % of the HCWs said they were aware of training offers for the management of multidrug-resistant organisms in their institution. They did know about possible aids to back-friendly working, although gaps in knowledge were apparent. Only 59 % of respondents knew that care utensils should preferably be stored at working height so as to reduce awkward body postures.

**Conclusions:**

Employees in elderly care are well informed about the range of precautionary occupational medical examinations and take advantage of this offering. Questions in the survey regarding hygiene management were answered in a competent manner. On the other hand some gaps in the knowledge about infection prevention and occupational safety became apparent. Differences between qualified and unqualified participating professionals occurred only in the knowledge of infectious diseases and pathogens and the associated path of infection. The extent to which training can help to improve infection prevention and occupational health and safety should be investigated.

## Background

Healthy employees are the most important resource for a healthy business. The implementation of measures to ensure occupational safety and the improvement of safety and health protection for employees is covered by the German Occupational Health and Safety [1].

The purpose of the Occupational Health and Safety Act [1] and its regulations (i.e., Ordinance on Biological Substances [2] is the health improvement of all employees in Germany. Especially in professional sectors like care work, a preservation of the employee’s health in the long run becomes increasingly important. As a result of demographic changes in Germany, the number of people in need of care will increase significantly in the years ahead. According to Hackmann [3] there will be approximately 4.4 million nursing cases by 2050. In other words, the proportion of individuals needing professional nursing care will increase by 270 %. In future, care will be provided less frequently by families and increasingly by professional institutions such as nursing homes and home care services. This means that an additional five hundred thousand healthcare workers (HCW) will be needed in the care sector [4].

Moreover, extending the length of time that is spent in a profession in a working life could help to meet the additional need alongside recruiting and training new healthcare staff. The average period worked by HCWs in elderly care is only 8.4 years to date, which is less than in medical healthcare and nursing [5]. The risk of HCWs in elderly care quitting their jobs prematurely is higher than in other fields [6]. People often leave the profession for “family” and “health” reasons. The health aspects could be countered by training and education in health protection and prevention. Moreover, general working conditions must be improved in order to prevent people from leaving the profession because of low income, physical workload, lack of flexible working hours, stress at work, retirement expectations and lack of recognition of the work accomplished by the profession [7]. Musculoskeletal disorders are the most common reasons for incapacity to work. The prevalence of back complaints in the population of HCWs for the elderly is almost 50 % [8]. The proportion of HCWs with an increased risk of long-term work incapacity due to musculoskeletal disorders has been estimated at 30 % [7]. Improved occupational safety and health protection for elderly-care workers could therefore make an important contribution toward prolonging the time people spend in the profession.

In 2012, HCWs in elderly care reported 899 spinal disc–related disorders of the lumbar spine to the Institution for Statutory Accident Insurance and Prevention in Healthcare and Welfare Services (BGW) [9]. High pressure loads on spinal discs when moving patients are often the probable cause of these disorders [10]. However, the pressure load on spinal discs when moving patients can be reduced considerably by using various working techniques and aids [11]. In addition to pressure loads, awkward body postures are common in elderly care [12]. These can be reduced considerably by adopting ergonomic measures such as raising bed heights [13].

Even more frequent than spinal disc–related disorders were reports of skin diseases (1,738 reports) and infections (1,250 reports) by geriatric HCWs [9]. The number of blood-borne virus infections has declined in recent years [14]. However, the number of tuberculosis infections reported has increased, as has the number of reported cases of methicillin-resistant *Staphylococcus aureus* (MRSA) colonization or infection [15]. In Germany, the risk of tuberculosis increases with age, so geriatric HCWs run a higher risk of infection [16]. The same applies to the risk of MRSA colonization, which is twice as high for nursing staff as for other healthcare staff [17].

The number of skin disease cases reported in elderly care is still high. The proportion of employees who have to quit the profession on account of a skin disease has fallen considerably thanks to the success of secondary individual prevention [18]. Nevertheless, skin diseases can result in long periods of absence. A controlled study showed that workplace seminars could lead to prevention of or improvement in skin-disorders [19].

Therefore, along with preventing skin diseases, occupational health and safety for HCWs in elderly care should concentrate on infection precautions and musculoskeletal disorders, an important task that can be covered, for instance, by occupational physicians. However, apart from medical attention, the employees’ ability to maintain their own health and avoid work-related health risks is a major prerequisite for successful occupational health and safety.

The aim of our study was to examine the employees’ knowledge of occupational health and safety issues and to determine differences between the participating professional groups.

## Methods

### Study population

In September and October 2012 we selected a convenience sample of the geriatric residential homes and their employees in the Schwerin area of Mecklenburg-Western Pomerania, Germany. All employees work for one employer in nine residential and two semi-residential nursing homes and for one homecare service. These institutions have a total of 820 residential places. We surveyed all employees in the institutions with direct contact with residents at work. A total of 473 employees received a questionnaire about infection prevention and occupational safety in elderly care. In order to preserve anonymity, no information on the study participants’ own residential areas was collected.

The institutions were recruited by presenting the project in person to the company’s board of directors. Participation in the study was voluntary. The questionnaire was accompanied by a letter explaining data privacy.

### The survey instrument

Based on the Occupational Health and Safety Act [1] the Ordinance on Biological Substances [2] regulations for precautionary medical examinations [20] and recommendations of Germanys central federal institution for disease control and prevention [21], we developed the questionnaire that was to be filled in by the employees themselves. Along with questions on socio-demographic data, it contains questions on the place of work, multidrug resistant organisms (MDRO) and infectious diseases. The closed questions have two (yes, no) or three (yes, no, not known) answer categories, or permitted multiple responses. Part A of the questionnaire, “Personal information about you”, solicits socio-demographic data such as gender, age, professional status and experience in (elderly) care work. Part B “General information on your workplace in respect of occupational health and safety” asks about the availability of an occupational physician at the workplace, implementation of a risk assessment and about preventive occupational healthcare and facilities that support back-friendly working. Part C “Infection control at your place of work” contains questions on hygiene training, a hygiene plan and on indications for and approaches to hand disinfection. The fourth set of questions, Part D “Multidrug resistant organisms in elderly care”, deals with training sessions on MDROs, the preventative measures required and with personnel screening. Part E “Infectious diseases: modes of transmission and protective measures” required multiple answers in its two sections. In the first table the participants are asked to identify the transmission modes of seven different infectious diseases or pathogens (Table 4). In the second table, respondents are asked to mark appropriate infection prophylaxis measures for three paths of infection (Table 5).

Table 4 suggests 28 possible answers, of which 12 are correct. Table 5 has 27 possible answers, of which 18 are correct and 9 either wrong or correct only in special situations. The number of correct answers is presented in Table 6.

A respondent with at least 10 out of 12 correct answers in Table 4 was assessed as having good knowledge, while a score of 8 to 9 was deemed satisfactory, 6 to 7 adequate, and fewer than 6 correct answers inadequate. Knowledge was also rated as inadequate if there were more wrong answers than correct ones.

Respondents with a score of at least 15 out of 18 possible correct answers in Table 5 were rated as having a good knowledge, while the knowledge of those with 13 to 14 was assessed as satisfactory, 11 to 12 as adequate and of those with fewer than 11 correct answers as inadequate.

The WHO lists five typical indicators for hand disinfection [22]. We added a sixth (wrong) indication to this list and asked participants to state which situation typically requires hand disinfection. The questionnaire also contains six images of hand disinfection procedures. Four images show a correct procedure (images 2, 3, 4 and 6) and two an incorrect procedure (images 1 and 5, Fig. 2).

Nine measures have been specified for dealing with MDROs, of which the German Recommendations suggest six as obligatory [21]. Three measures are not wrong, but not obligatory and usually unnecessary. The following measures should always be adopted when dealing with MDROs: strict adherence to hygiene regulations, hand disinfection, use of protective clothing (disposable gloves and nose and mouth protection where contact with infectious material is possible, protective gowns in the event of close contact with MRSA-positive residents) [21]. The following are useful in certain situations, but not always necessary: displaying warning notices on residents’ rooms, strict isolation of infected persons, wearing of protective goggles.

The questionnaire was tested by some nurses in elderly care. To increase the response rate we reminded all participants after four weeks to complete and return the questionnaires.

The questionnaires were analysed descriptively using the SPSS statistics program (Version 21). For the comparison of occupational groups the classification in qualified geriatric and medical HCWs and care assistants was used. Differences between professional groups were ascertained by means of the Chi-square test or Fisher’s exact test. The level of significance was specified as *p* <0.05.

### Ethical consideration

All data in this trial was collected, analyzed and disclosed anonymously, following the terms of the Hamburg Medical Association and the Data Protection Act of the City of Hamburg (HmbDSG).

## Results

Of the 473 questionnaires sent out, 132 were completed and returned, equivalent to a response rate of 28 %. The study population is described in Table 1. Eighty-four per cent of the participants were women. The largest age group was those under 30 years (29 %), followed by the 40- to 49-year-olds (28 %). Thirty-six per cent of respondents were qualified geriatric HCWs, 16 % medical HCWs, and 27 % were care assistants. The majority of respondents had up to 5 years’ experience of elderly-care work (36 %), and 17 % more than 20 years’ experience.

**Table 1:** Description of study population

	N	%
Total	132	100
Gender		
Female	111	84.1
Male	21	15.9
Age		
Under 30 years	38	28.8
30–39 years	26	19.7
40–49 years	37	28.0
50–59 years	28	21.2
Over 59 years	3	2.3
Professional status		
Geriatric HCWs (qualified)	48	36.4
Medical HCWs (qualified)	21	15.9
Care assistants	35	26.5
Others	28	21.2
Years in elderly care		
0–5 years	48	36.4
6 –10 years	25	18.9
11–15 years	18	13.6
16–20 years	18	13.6
>20 years	23	17.4

### Workplace and occupational health and safety in the institution

Eighty-three per cent of respondents said that regular precautionary checks and vaccinations against infectious diseases were offered (Table 2). Of those, 93 % took advantage of such offers. Three quarters (74 %) of respondents felt well informed about precautionary checks and vaccinations against infectious diseases. Eighty per cent of respondents knew the on-site occupational physician. Just over half of employees (51 %) did not know whether a risk assessment had been carried out at their workplace. Asked whether a needle-stick injury was followed up with a check by the occupational physician, 66 % chose “Yes”. Asked the main things to which attention should be paid to assist back-friendly moving and handling of residents, 99 % ticked “Use of aids” and “Adjusting bed to working height”, while 84 % saw “Working in pairs” and 59 % “Keeping care utensils at working height” as important.

**Table 2:** Knowledge of healthcare staff about occupational safety in their institution

	Yes	No	Not known	Total
	N (%)	N (%)	N (%)	N (%)
Occupational physician on site	106 (80.3)	25 (18.9)	1 (0.8)	132 (100.0)
Risk assessment conducted	48 (36.4)	16 (12.1)	68 (51.1)	132 (100.0)
Regular precautionary checks and vaccinations on offer	110 (83.3)	11 (8.3)	11 (8.3)	132 (100.0)
Precautionary check accepted	102 (92.7)	8 (7.3)	-	110 (83.3)
Informed about precautionary check	98 (74.2)	34 (25.8)	-	132 (100.0)
Occupational physician consulted after needle-stick injury	87 (65.9)	45 (34.1)	-	132 (100.0)

### Infection control

**Fig. 1:**
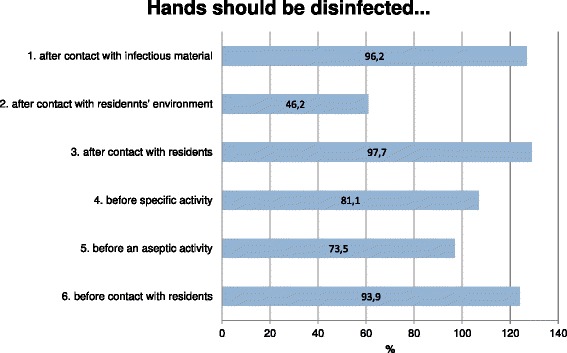
When should hands be disinfected: 5 Indications for hand disinfection [17]. Numbers 1–3 and 5–6 are correct

Asked whether there were regular infection control training offers, 92 % of respondents chose “Yes” (Fig. 1). All respondents (100 %) agreed that the hygiene plan was available to all employees at any time. Asked when hands should be disinfected, 98 % opted for “After contact with residents”, 96 % for “After contact with potentially infectious material”, 94 % for “Before contact with residents”, 74 % for “Before an aseptic activity” and 46 % “After contact with patients’ immediate surroundings”. Eighty-one per cent ticked “Before septic activities”, the only answer that does not appear in the WHO list of five indications for hand disinfection [22] (Fig. 1). All five correct indications were selected by 42 % of respondents, while 21 % selected three indications, and 7 % only two (no table).

**Fig. 2:**
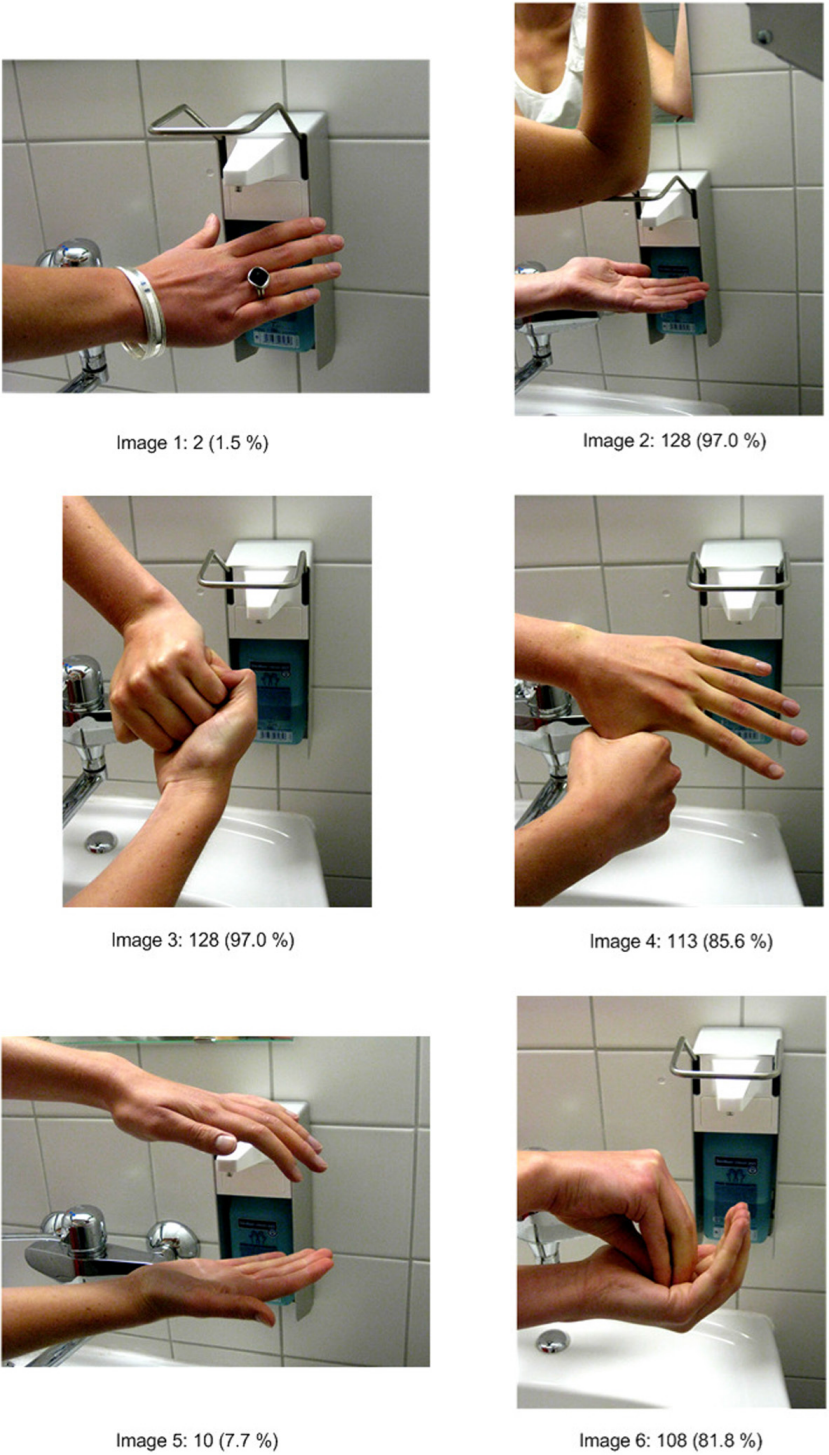
Which steps form part of correct hand disinfection? Multiple answers possible. Images 2, 3, 4, 6 are correct.

Regarding the images of correct procedures of hand disinfection, 97 % chose Image 2 and/or Image 3, 86 % Image 4 and 82 % Image 6, while approximately 2 % of respondents classed Image 1 and around 8 % Image 5 as correct procedures (Fig. 2).

### Multidrug resistance organisms in elderly care

Sixty-six per cent of respondents said that MDROs training took place; 92 % agreed that employees were informed if a resident had an MDRO infection, and 91 % stated that their institution had a standard procedure for dealing with MDRO infections. (Table 3).

**Table 3:** Dealing with multidrug resistant organisms (MDRO) in nursing homes for the elderly

	Yes	No	Not known	Total
	N (%)	N (%)	N (%)	N
MDRO training	86 (65.6)	14 (10.7)	31 (23.7)	131
Informed about MDRO cases	120 (91.6)	11 (8.4)		131
Personnel screened for MDROs	13 (9.9)	58 (44.3)	60 (45.8)	131
MDRO standard exists	118 (90.8)	12 (9.2)		130

**Fig. 3:**
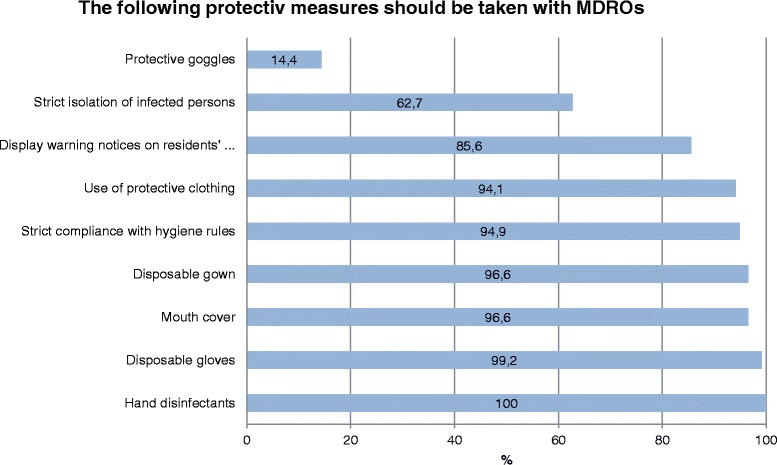
What protective measures are required for dealing with residents with an MDRO infection?

Regarding protective measures to be taken in such cases, all respondents chose “Hand disinfection”, 99 % selected “Disposable gloves”, and 97 % “Mouth protection” and/or “Disposable gowns” (Fig. 3). Fourteen per cent opted for “Protective goggles”. The six options that are always advisable were chosen by 76 % of participants. A further 11 % placed a cross by five of the six measures (no Table).

### Infectious diseases: transmission modes and protective measures

With these questions, employees were asked to match seven different infectious diseases or pathogens with their mode(s) of transmission. The study participants’ responses are shown in Table 4. The proportion of correct answers ranges from 94 % for droplet infection in the case of influenza to 29 % for droplet infection with adenoviral conjunctivitis. More than 80 % of respondents marked a cross by seven of the 12 correct answers. In terms of school-type grades for the quality of knowledge, 23 % of participants had good knowledge and 33 % had satisfactory knowledge (Table 6).

**Table 4:** Employees’ knowledge of infectious diseases and/or pathogens and their paths of infections

Infections^a^	Paths of infection				
	Contact/smear infection (faecal/oral)	Droplet infection (aerogenous)	Food and water (alimentary)	Blood (haemato-genous)	Total
	N (%)	N (%)	N (%)	N (%)	N
Conjunctivitis (Adenoviral)	**100 (88.5)**	**33 (29.2)**	2 (1.8)	2 (1.8)	113
Flu (Influenza)	**39 (32.8)**	**112 (94.1)**	5 (4.2)	1 (0.8)	119
Hepatitis (B and C)	29 (24.4)	16 (13.4)	17 (14.3)	**107 (89.9)**	119
MRSA	**102 (84.3)**	**83 (68.6)**	6 (5.0)	35 (28.9)	121
Norovirus infection	**96 (81.4)**	**53 (44.9)**	39 (33.1)	5 (4.2)	118
Salmonella infection	**61 (49.6)**	13 (10.6)	**107 (87.0)**	3 (2.4)	123
Lung tuberculosis	23 (19.7)	**96 (82.1)**	9 (7.7)	32 (27.4)	117

^a^Multiple answers possible, correct answers are bold

Table 5 shows the responses on protective measures by mode of transmission. The proportion of correct answers ranges from 100 % for hand disinfection to prevent contact and smear infection to 34 % for protective vaccination against contact and smear infection. More than 80 % of participants who answered chose eleven of the 18 correct answers, while 32 % had a good knowledge and 33 % had a satisfactory knowledge of protective measures (Table 6).

**Table 5:** Employees’ knowledge of protective measures by modes of transmission

	Mode of transmission^a^			
	Contact/smear infection (faecal/oral)	Droplet infection (aerogenous)	Blood (haemato-genous)	Total
	N (%)	N (%)	N (%)	N
Hand disinfection	**121 (100.0)**	**75 (62.0)**	**69 (57.0)**	121
Protective vaccination	**39 (34.2)**	**89 (78.1)**	**81 (71.1)**	114
Mouth and nose cover	58 (47.9)	**115 (95.0)**	17 (14.0)	122
Single room	**92 (83.6)**	**86 (78.2)**	27 (24.5)	110
Disposable glove	**119 (96.7)**	94 (76.4)	**113 (91.9)**	123
Protective goggles	37 (39.4)	**84 (89.4)**	26 (27.7)	94
Disposable materials	**113 (93.4)**	**97 (81.8)**	**99 (80.2)**	121
Protective gown	**110 (94.0)**	**88 (75.2)**	71 (60.7)	117
Surface disinfection	**119 (97.5)**	105 (86.1)	101 (82.8)	122

^a^Multiple answers possible, correct answers are bold

**Table 6:** Employees’ knowledge of infectious diseases and/or pathogens and their paths of infection, and employees’ knowledge of protective measures by mode of transmission, classified and analysed

Knowledge of pathogens**	Geriatric HCWs (qualified)	Medical HCWs (qualified)	Care assistants	Social service, supervisors, trainees, community service	Total
	N (%)	N (%)	N (%)	N (%)	N (%)
Good	12 (25.0)	9 (42.9)	6 (17.1)	3 (10.7)	30 (22.7)
Satisfactory	17 (35.4)	7 (33.3)	11 (31.4)	9 (32.1)	44 (33.3)
Adequate	14 (29.2)	5 (23.8)	7 (20.0)	12 (42.9)	38 (28.8)
Inadequate	5 (10.4)	0 (−−)	11 (31.4)	4 (14.3)	20 (15.2)
Protective measures					
Good	16 (33.3)	10 (47.6)	9 (25.7)	7 (25.0)	42 (31.8)
Satisfactory	15 (31.3)	6 (28.6)	14 (40.0)	8 (28.6)	43 (32.6)
Adequate	8 (16.7)	2 (9.5)	2 (5.7)	6 (21.4)	18 (13.6)
Inadequate	9 (18.8)	3 (14.3)	10 (28.6)	7 (25.0)	29 (22.0)
Total	48 (100.0)	21 (100.0)	35 (100.0)	28 (100.0)	132 (100.0)

**P = 0.023

Here, no significant difference occurred between the professional groups. However, the only statistically significant difference between qualified and assistant staff showed in the knowledge of infection paths. Almost one third (31 %) of care assistants had inadequate knowledge about protective measures (Table 6).

## Discussion

No previous questionnaire-based survey of geriatric HCWs’ knowledge of infection prevention and occupational health and safety had been undertaken, making this the first study to explore the subject. The available literature tends to refer to existing knowledge about how to improve the care of patients [21]. Consequently, despite the small sample size and the moderate response rate of 28 %, our study represents the first indication of geriatric HCWs’ degree of knowledge. The participating HCWs answered questions regarding infection control in a competent manner. On the other hand our survey showed some gaps in the knowledge about infection prevention and occupational health. Differences between qualified and unqualified participating professionals occurred only in the knowledge of infectious diseases.

Occupational-medical coverage, including preventive and precautionary measures are one of the major elements of the German Occupational Health and Safety Act [1]. While most employees were acquainted with the occupational physician on site, around half did not know whether a risk assessment had been conducted at their workplace. The frequency of risk assessments in nursing homes for the elderly and whether employees are adequately informed about them should be investigated. On a positive note, 90 % of those who were aware of the precautionary offers available also utilised them. According to Kromark et al. [23] there is no difference between older and younger employees as regards their participation in preventive measures. Sixteen per cent of respondents did not know that regular precautionary checks and vaccinations were on offer.

Back-friendly working is another important aspect of occupational health. Looking after elderly, immobile residents places great physical strain on healthcare staff, who often work in awkward body postures [24]. Older geriatric HCWs are more likely to suffer from complaints of the cervical spine and lumbar spine than their younger colleagues [23]. Dulon et al. [25] determined a prevalence of 48 % for lumbar-spine complaints and of around 17 % for complaints concerning the neck and shoulder area [25]. They concluded that increasing age and career length in the nursing profession–along with earlier back problems and psychological stress at work–were significant risk factors. Nearly all respondents chose “Adjusting beds to working height” and “Use of aids” as important for practising back-friendly working. Next came “Working in pairs”, though this can hardly be guaranteed because of the staffing situation. Only 60 % regarded “Keeping care utensils at working height” as important. This highlights a gap in knowledge that should be dealt with in training courses. Employees in nursing homes for the elderly have a significant advantage over outpatient HCWs in terms of the availability of aids and help from colleagues. However, orthopaedic complaints were more frequently found among employees in residential institutions [8]. With any measures, however, it is important to ask how theoretical knowledge is put into practice. Too few staff, too much work, too little time, an absence of aids, aids that are difficult to use, or possibly even staff complacency can be reasons these important measures for back-friendly working are not or only partly adopted. In order to prevent existing especially musculoskeletal complaints from becoming chronic – thereby leading employees to quit the profession – precautionary measures are needed [26].

An increase of multiresistant pathogens in hospitals and care facilities for the elderly requires the awareness of and the adherence to infection control guidelines. Appropriate knowledge is therefore of central significance for the prevention of infection. All respondents agreed that the hygiene plan was accessible to all, reflecting results from a study by Peters et al. [27]. They performed a cross sectional study on infection control in residential geriatric nursing facilities in Germany 2012. The questionnaire recorded important parameters of hygiene, resident and staff protection and actions in case of existing MDROs. Most of the residential geriatric nursing homes had standards for MDROs and regular hygiene training for staff. The facilities provided adequate protective clothing, affected residents are usually isolated and hygienic laundry processing conducted [27]. A further important aspect is the knowledge about correct hand disinfection. It appears that most employees disinfect their hands “After contact with residents”, “After contact with potentially infectious material” and “Before contact with residents”. They tend to see the risk of infections in activities involving direct contact with residents or patients rather than from the environment. Respectively, further training in this area seems advisable. However, one must also question the extent to which these indications are acknowledged in practice. Aiello et al. [28] showed that employees’ knowledge of hygiene measures and infection sources led them to carry out preventive measures, but that they did not always know the correct way of doing so. According to a study by Ashraf et al. [29], reasons employees do not follow guidelines when carrying out hygiene measures include a lack of equipment such as alcohol-based disinfectants in the vicinity of employees in long-term care facilities. The same study also found that although the majority of employees were familiar with the existing hygiene guidelines and considered them important, only one third answered the relevant questions correctly. The authors came to the conclusion that training courses should be used to help employees understand existing hygiene guidelines so as to eliminate obstacles to applying them. Sound knowledge contributes toward better compliance and thus more effective protection. However, 31 % of respondents said that despite having guidelines they would not change their hand hygiene practices [29].

Larson et al. [30] found that various methods of influencing employees’ hand hygiene, such as feedback, training courses or provision of better technical equipment, had only a minimal long-term effect on the frequency of hand washing. Yet 40 % of healthcare staff took the view that “six to ten hand-cleaning units per hour” were necessary to avoid nosocomial infections. In fact, those carers often washed their hands less than five times per hour [31]. The study by Alvaran et al. [32] showed that although HCWs in elderly care had more theoretical knowledge, care assistants had better subjective knowledge of hygiene techniques. However, it was unable to show an association between the mention of more frequent hand hygiene and the current degree of knowledge or a positive attitude to hand hygiene [32]. Our study did not examine whether knowledge is relevant to daily behaviour. Parmeggiani et al. [33] found in a questionnaire-based survey of emergency departments in Italian hospitals that, despite good knowledge and a positive attitude toward the usual ways of protecting against nosocomial infections, the level of compliance with those measures was rather low.

In Germany the recommendations [21] for “Infection Prevention in Long-term Care Facilities” are regarded as an important model for dealing with and preventing infections. The recommendations also include measures relating to residents, personnel, visitors and the surroundings in the event that residents are infected with MDROs, especially with MRSA. As regards MDROs in elderly care in our study, approximately two thirds of respondents were aware of training offers, while most said that they were informed if a resident had an MDRO infection. This information should always be provided so that healthcare staff can adapt to the new, potentially infectious situation. Residential nursing homes for the elderly should also inform the hospital if one of their residents with a (known) MDRO infection has to be transferred to hospital. However, only 10 % of employees said that nursing homes screened their personnel if there were frequent cases of MDRO infection. Employees can be MDRO infection carriers, and this would be a way of identifying possible carriers and of preventing further infections both among residents and staff.

Sowirka et al. [34] revealed that twenty-nine per cent of healthcare staff in a nursing home were tested positive for *Staphylococcus aureus* in a non-epidemic situation, and 14 % of these were MRSA carriers. No significant association was found between MRSA carriage and age, length of service or professional position. However, another study found that women were more frequently affected than men [35]. One study showed that bags and work clothing were especially heavily contaminated with MRSA; almost all of respondents marked “Use of protective clothing” as a possible protection against this [36]. The German recommendations [21] advise that disposable gloves and a mask for the mouth and nose be worn in the event of possible contact with infectious materials, and gowns in the event of close contact with MRSA-positive residents. It also recommends disinfecting the hands after direct contact with residents, after removing protective clothing and on leaving the patient’s room. Our results show that nearly all of the HCWs agreed with the use of disposable gloves and with wearing a mouth cover and/or disposable gown. The employees regarded the use of hand disinfectants as an important measure for protection from MDRO infections and as an important way of avoiding transmission. Two thirds opted for strict isolation of infected persons. Since this requires appropriate rooms, it is hard to implement in some nursing institutions for the elderly, especially when they are fully occupied and have multi-bed rooms. According to the recommendations, isolation of MRSA-positive residents in nursing homes is not obligatory. Cohort isolation of a group of MRSA-positive residents may be considered. It must also be ensured that there is no risk of transmission to or infection of MRSA-negative fellow residents, for example, through open wounds or devices [21]. In the event of isolation, it would be desirable to have additional personnel to cope with the more demanding requirements. According to Furuno et al. [37] the majority of HCWs see isolation as the greatest aid to reducing the transmission of resistant pathogens. Yet one must consider the negative consequences of isolation, including adverse psychological effects such as depression and anxiety [38]. Whether these protective measures should be adopted in the event of MDRO incidences requires investigation.

In assessing the study findings it should be borne in mind that the method used was subjective (employee survey in writing). Thus the answers to questions depend both on the respondents’ state of mind at the time and on their individual perception and way of thinking. Self-reported information may entail distortions in response behaviour, e.g., along the lines of what is socially desirable. The employees who completed the questionnaire may be those who were already intensely preoccupied with the topics of hygiene and occupational health and safety or who had experienced problems in this connection at the workplace. Moreover, the questionnaire is a measuring instrument developed specially for this survey and used for the first time. Since the response rate was only 28 %, the results should be interpreted with caution. The survey was not directed to scrutinize why existing knowledge is not being applied and it can only be assumed that the discrepancy between knowledge and application of hygiene standards is often influenced by lack of time and staff at the workplace.

In addition, the employees surveyed all worked for the same employer, so the findings may not be transferable to other institutions. In this respect, this study should be seen as an explorative study that attempts for the first time to record what HCWs in elderly care know about infection prevention and occupational health and safety.

## Conclusion

Our study concludes that geriatric HCWs in our sample are well informed about the range of preventive occupational medical examinations and answered questions regarding infection control in a competent manner. No significant differences between qualified and unqualified participating professionals occurred. On the other hand there are some gaps in geriatric HCWs’ knowledge of occupational health and safety and infection control. Further efforts to improve employees’ knowledge are required. Systematic investigation of the knowledge, attitude and associated behaviour of HCWs in care of the elderly is needed, so as to improve understanding of how to positively influence employees’ behaviour.

## Abbreviations

BGW: Statutory Accident Insurance and Prevention in Healthcare and Welfare Services
HCW: Healthcare workers
MRSA: Methicillin-resistant staphylococcus aureus
MDRO: Multidrug resistant organism
